# Assessing Avascular Necrosis Risk and Outcomes After Open Reduction for Developmental Dysplasia of the Hip in Children

**DOI:** 10.7759/cureus.75808

**Published:** 2024-12-16

**Authors:** Mohamed Zamzam, Khalid A Bakarman, Abdulrahman A Alaujan, Abdelrafour Houdane, Yazeed A AlKhayyal, Homoud Al Zaid, Abdulmonem Alsiddiky, Kholoud Alzain, Fahad Alhuzaimi, Fayez G Aldarsouni

**Affiliations:** 1 Orthopedics, King Saud University, Riyadh, SAU; 2 Pediatrics, King Abdullah Specialized Children's Hospital, Riyadh, SAU; 3 College of Medicine, Alfaisal University, Riyadh, SAU; 4 General Surgery, King Khalid University Hospital, Riyadh, SAU; 5 Radiology, King Faisal Specialist Hospital and Research Centre, Riyadh, SAU; 6 Orthopedic Surgery, King Saud Medical City, Riyadh, SAU; 7 General Surgery, Prince Sultan Military Medical City, Riyadh, SAU; 8 Trauma Surgery, King Saud Medical City, Riyadh, SAU

**Keywords:** avascular necrosis, ddh, developmental, dysplasia, hip, risk factors

## Abstract

Background

Avascular necrosis (AVN) of the femoral head is a serious complication after surgical treatment of developmental dysplasia of the hip (DDH). The main objective of this study is to identify the incidence of AVN and to define AVN risk factors. The study also aims to identify the effects of AVN and other factors on final clinical and radiological outcomes.

Methods

All patients in the study were managed according to King Saud University protocol for surgical treatment of DDH. Occurrence of AVN was reported. All affected hips were evaluated clinically and radiologically after six and 12 months and at final follow-up. Significant variables from the univariate analysis were entered into multivariate regression models.

Results

A total of 131 patients were included in the study involving 189 hips with DDH. AVN was diagnosed postoperatively in 23 (12%) hips. In the multivariate regression analysis, the presence of AVN (p=0.002), prolonged casting (p=0.016) and the need for a second surgery (p=0.029) were strong determinants of unsatisfactory clinical outcomes. Worse radiological outcomes were significantly associated with the presence of AVN (p=0.002), previous trials of closed reduction (p=0.032) and the need for a second surgery (p=0.013). In the last multivariate regression model, a previous trial of closed reduction (p=0.001), absence of an ossified femoral head (OFH) (p=< 0.001), prolonged casting (p=0.038), and the need for second surgery (p=0) were strong risk factors for AVN.

Conclusions

The current study highlighted the factors responsible for AVN occurrence after open DDH reduction. The factors which are strong determinants of unsatisfactory clinical and radiological outcomes were identified. Overcoming these factors is mandatory to improve the results.

## Introduction

The goal of successful management of developmental dysplasia of the hip (DDH) is to achieve and maintain a concentrically reduced hip, and to end with a painless mobile radiologically appearing normal hip without occurrence of complications [1,2]. Many complications could arise after treatment of DDH, however the most serious complication is avascular necrosis (AVN) of the femoral head because of its high incidence and undesirable consequences [3]. There is considerable concern about the risk of iatrogenic AVN and its long-term impact on both patient function and outcomes [4-8].

AVN is considered one of the most devastating complications that could occur post-treatment [3,9]. AVN of the femoral head can lead to many adverse sequelae, such as acetabular dysplasia with persistent subluxation or redislocation, limb length discrepancy, and joint incongruities that render the patient more prone to developing early-onset osteoarthritis. In most cases, AVN subjects the patients to more surgeries which ultimately negatively affects the patient’s quality of life [10,11].

Due to the nature of AVN after surgical treatment of DDH and its consequences, it is imperative to identify relevant risk factors in an effort to circumvent and avoid its detrimental adverse effects. Various studies explore the myriad of risk factors that increase the risk of AVN in DDH treatment but no clear consensus has been reached.

Multiple risk factors have been identified such as forced reduction, cast length or positioning, age at surgery, high dislocation, and surgical technique [10,12]. It has also been suggested in the literature that the presence of an ossific nucleus is associated with a decreased incidence of AVN [13,14]. However, other studies concluded that an ossific nucleus does play any role in AVN occurrence [3,12,14,15]. A systemic review by Sankar et al. also showed that presence of an ossific nucleus and age at reduction were both not associated with the presence of AVN [8].

Some studies show that successful closed reduction has a lower AVN rate than open reduction [1,16]. However, a cohort study by Alfaleh et al. demonstrated a significantly higher rate in AVN in patients who underwent closed reduction and hip spica compared to other treatments [3]. In another study, non-operatively treated DDH patients had higher rates of AVN compared to the group who received open reduction and Salter's innominate osteotomy [17].

With contradictory findings in the literature, more research effort is required to better understand the risk factors involved. The aim of this study is to identify the incidence of AVN after open reduction for DDH and to define AVN risk factors that predispose to its occurrence. The study also aims to determine the effects of AVN and other factors on patients’ clinical and radiological outcomes.

## Materials and methods

After obtaining approval from King Saud University Institutional Review Board (E-21-6239), a retrospective cohort study was conducted at King Saud University Hospital where all patients under six years of age who underwent open reduction with or without acetabuloplasty and femoral shortening as a treatment for DDH during the period from January 2010 to January 2020 were reviewed. The patients’ demographic, clinical, and radiological data were collected from medical records. Deficient data were completed by calling the parents to bring their children to be reviewed in the clinic. Patients who had a neuromuscular or teratological dislocation, dislocations associated with infection, previous history of AVN, or patients missed during follow-up were all excluded from the study.

All patients in the study were managed according to the King Saud University protocol for surgical treatment of DDH. Open reduction (OR) is indicated in children older than six months of age after failure of closed reduction. When feasible, either Pemberton or Dega pelvic osteotomies are the routine acetabuloplasty (AP) of choice that is performed with OR based on the status of the posterior acetabular wall and/or surgeon’s preference. Femoral shortening (FS) is a routine procedure in all patients older than two years of age and sometimes is performed in younger patients with very high-riding dislocations or when intraoperative reduction is very tight or impossible without femoral shortening. After surgery, hip spica was applied in all patients for six weeks and followed by a "broomstick cast" for three to four weeks. In certain situations, due to an unstable hip or delayed union of the pelvic osteotomy site, discontinuing the cast was delayed. Prolonged casting was considered when the cast stayed more than 90 days. After cast removal, all patients have been followed for a minimum of two years, with an average follow-up of 35.8 months (SD = 10.4) and a range of 24-74 months. All patients were reviewed every three months during the first postoperative year and subsequently every six months. Follow-up protocol could be altered if needed particularly with the presence of complications.

Collected data were categorized as preoperative, intraoperative, or postoperative variables. Patients were classified according to their ages into three groups which reflects the possible need for acetabuloplasty, femoral shortening, or both with open reduction. The first are those less than two years, the second are two to three years, and the third are more than three years of age. Early complications were reported for each case, while delayed or late complications were considered during follow-up by clinical and radiological assessment. The occurrence of AVN was reported individually and according to the classification of Kalamchi and MacEwen [5], were classified into four groups and referred to as Kalamchi groups I, II, III, and IV.

All affected hips were evaluated clinically and radiologically after six and 12 months. Clinical evaluation included the appearance of the scar, the presence of pain and tenderness, the range of movement, any limb-length discrepancy, and the Trendelenburg sign if applicable. Deviation from normal of any of these criteria was considered unsatisfactory. Unsatisfactory radiological assessment included lateralization or re-dislocation of the femoral head, persistent acetabular dysplasia after acetabuloplasty, or non-union at the pelvic osteotomy site.

At the final follow-up, all hips were assessed clinically according to Berkeley et al. [18] where excellent was defined as a painless stable hip without a limp or positive Trendelenburg sign, with more than 15° of internal rotation and otherwise normal motion; good; a painless stable hip with a slight limp or decreased range of motion and a negative Trendelenburg sign, fair; a positive Trendelenburg sign, minimum pain, and moderate stiffness; and finally poor; patient in significant pain. Excellent and good were considered satisfactory clinical results, while fair and poor were considered unsatisfactory. Severin’s criteria [19] were used to assess the final radiological outcome. Those hips with Severin I were considered normal (Category 1), II and III were considered to have acceptable results (Category 2), while IV, V, or VI were considered unacceptable (Category 3).

Demographics and clinical characteristics were reported as means with standard deviations (SDs), medians with interquartile ranges (IQRs), ranges, or frequencies with percentages (%). Comparative analyses were done using Student’s t-test for continuous data (if normally distributed), or the Mann-Whitney U test. The chi-square test was used to compare categorical variables.

The study cohort was grouped into AVN vs non-AVN groups. Univariate analysis was carried out to identify significant variables associated with AVN.

Significant variables from the univariate analysis were entered into multivariate regression models. Clinical outcome (Unsatisfactory vs. Satisfactory) was used in a logistic regression model with a binary response. Furthermore, radiological outcome (Category 1 vs. Category 2 and Category 3) was used in another logistic regression model. Finally, AVN (Yes vs. No) was used in a logistic regression model with a binary response. Multicollinearity was measured using the variance inflation factor (VIF). In the case of high multicollinearity, one or more variables were excluded from the model. Odds ratio (OR) and 95% confidence intervals (95%CIs) were reported for the regression models.

SPSS software version 23 (IBM Corp., Armonk, NY, USA) was used for data entry and analysis. All analyses were carried out at a 5% two-sided significance level.

## Results

A total of 131 patients were included in the study which involved 189 hips with DDH. Median age at presentation for the overall cohort was 18 months (IQR, 15-25) and primarily comprised of females (88%). The median age at surgery was 27 months (IQR, 18 - 22). Baseline characteristics are presented in Table 1.

**Table 1:** Baseline characteristics (n = 131) IQR: Interquartile range, SD: Standard Deviation

Characteristic	-	-
Age at presentation (months)	Median (IQR)	18 (15 – 25)
-	Mean (SD)	21.5 (12.3)
-	Range	(1.46 – 70.0)
Age at surgery (months)	Median (IQR)	27 (18 – 22)
-	Mean (SD)	24.6 (12.0)
-	Range	(5.73 – 72.0)
-		N (%)
Sex	Male	16 (12)
	Female	115 (88)
Diagnosis	Right	31 (24)
	Left	42 (32)
	Bilateral	58 (44)

Out of all hips included in the study, 112 (59%) underwent OR + AP, 40 (21%) had OR + AP + FS, and 37 (20%) had only OR surgery. AVN was diagnosed postoperatively in 23 (12%) hips. AVN as a complication was considered and analyzed individually. A total of 35 hips had experienced one or more post-operative complications either due to the presence of AVN or not. These complications included seven superficial wound infections, 32 stiffness, 14 femoral head lateralization, five re-dislocations, nine persistent acetabular dysplasias, three displacements at femoral osteotomy sites, and three significant limb length discrepancies (LLD) that required further treatment. Superficial wound infections were treated by wound care only for six hips and an antibiotic was needed according to culture and sensitivity results for one hip. One or more further surgeries were performed for 23 hips due to complications, which included seven arthrographic assessments and/or manipulation under anesthesia for stiffness or femoral head lateralization, seven re-open reductions for redislocation or lateralization, six revision acetabuloplasties for persistent acetabular dysplasia, three for re-fixing the femoral osteotomy and three to equalize LLD. Only two hips of those who had post-operative complications other than AVN resulted in unsatisfactory clinical outcomes at final follow-up and none of them resulted in poor radiological outcomes.

A significant number of hips in the AVN group had a previous trial of closed reduction or non-ossified femoral head (OFH) [13 (57%) and 17 (74%) respectively] compared to the non-AVN group (P < 0.001). On the contrary, there was no significant difference between AVN and non-AVN groups in terms of age at surgery (P = 0.58) (Table 2). Although the operation time for the AVN group seems longer than that for the non-AVN group, this difference was insignificant (P = 0.72). Likewise, no significant difference was found between the two groups in terms of surgical procedure used (P = 0.50) or the need for blood transfusion (P= 0.65) (Table 3).

**Table 2:** Comparison between non-AVN and AVN groups in terms of pre-operative variables AVN: Avascular Necrosis *: statistically significant p value

Variable		Non-AVN N = 166	AVN N = 23	P
-		Median (IQR)		-
Age at surgery (months)		22 (17 – 28)	22 (19 – 30)	0.58
-		N (%)		-
Previous trial of closed reduction	Yes	16 (10)	13 (57)	< 0.001*
	No	150 (90)	10 (44)	
Ossified Femoral Head (OFH)	Yes	140 (84)	6 (26)	< 0.001*
	No	26 (16)	17 (74)	

**Table 3:** Comparison between non-AVN and AVN groups in terms of intra-operative variables AVN: Avascular Necrosis, OR: Open Reduction, AP: Acetabuloplasty, FS: Femoral Shortening

Variable	-	Non-AVN n = 166	AVN n = 23	P
-		Median (IQR)		-
Operation time (min)	-	198 (156 – 298)	221 (170 – 327)	0.72
-		N (%)		-
Surgical procedure	OR	31 (19)	6 (26)	0.50
	OR + AP	98 (59)	14 (61)	
	OR + AP + FS	37 (22)	3 (13)	
Blood transfusion	Yes	23 (14)	4 (17)	0.65
	No	143 (86)	19 (83)	

Table 4 shows that the AVN group of hips had a significantly longer period of casting compared to the non-AVN group (P = 0.001). There was also a significant relation between the occurrence of AVN and the presence of other complications and the need for a second surgery (P = 0.001 and < 0.001 respectively). On the other hand, there were no significant differences regarding percentages of the need for physiotherapy (to treat stiffness of the affected hip) between AVN and non-AVN groups (P = 0.21).

**Table 4:** Comparison between non-AVN and AVN groups in terms of post-operative variables AVN: Avascular Necrosis *:statistically significant p-value

-	-	Non-AVN N = 166	AVN N = 23	P
-		Median (IQR)		-
Period of casting (days)	-	76 (68 – 90)	91 (75 – 115)	0.001*
-		N (%)		-
Period of casting	Prolonged	45 (27)	14 (61)	0.001*
	Normal	121 (73)	9 (39)	
Complications	Yes	25 (15)	10 (44)	0.001*
	No	141(85)	13 (56)	
Second surgery	Yes	14 (8)	9 (39)	< 0.001*
	No	152 (92)	14 (61)	
Physiotherapy	Yes	15 (9)	4 (17)	0.21
	No	151 (91)	19 (83)	

At six-month follow-up, there was no significant difference between AVN and non-AVN groups in regard to clinical and radiological assessment (P = 0.08 and 0.49, respectively). On the other hand, at 12-month follow-up, unsatisfactory clinical outcomes were significantly higher in the AVN group relative to the non-AVN group (P = 0.021) but there is no significant difference in regards to radiological outcomes (P = 0.49) (Table 5).

**Table 5:** Clinical and Radiological Outcomes at six- and 12-month follow-up for the study cohort according to the presence of AVN AVN: Avascular Necrosis *: statistically significant p-value

-	-	Non-AVN N = 166 n (%)	AVN N = 23 n (%)	P
6-month follow-up				
Clinical Outcome	Unsatisfactory	21 (13)	6 (26)	0.08
	Satisfactory	145 (87)	17 (74)	
Radiological Outcome	Unsatisfactory	3 (2)	0 (0)	0.49
	Satisfactory	163 (98)	23 (100)	
12-month follow-up				
Clinical Outcome	Unsatisfactory	16 (10)	6 (26)	0.021*
	Satisfactory	150 (90)	17 (74)	
Radiological Outcome	Unsatisfactory	3 (2)	0 (0)	0.49
	Satisfactory	163 (98)	23 (100)	

For clinical outcomes at the final follow-up, 30% of the AVN group had unsatisfactory results compared to 1% for the non-AVN group (P < 0.001). Furthermore, with respect to the radiological outcome at the final follow-up, 90% of the non-AVN group were in category 1 (normal hips according to Severin’s criteria) and 0% in category 3 (unsatisfactory radiological outcome) relative to 17% and 9% respectively in AVN group (P < 0.001) (Table 6).

**Table 6:** Clinical and Radiological Outcomes at final follow-up for the study cohort according to the presence of AVN AVN: Avascular Necrosis *: statistically significant p-value

-	-	Non-AVN N = 166 n (%)	AVN N = 23 n (%)	P
Clinical outcome	Unsatisfactory	2 (1)	7 (30)	< 0.001*
	Satisfactory	164 (99)	16 (70)	
Radiological outcome (Grades)	I	149 (90)	4 (17)	< 0.001*
	II	12 (7)	10 (44)	
	III	5 (3)	7 (30)	
	IV	0 (0)	1 (4.5)	
	V	0 (0)	1 (4.5)	
	VI	0 (0)	0 (0)	
Radiological outcome (Categories)	1	149 (90)	4 (17)	< 0.001*
	2	17 (10)	17 (74)	
	3	0 (0)	2 (9)	

At the final follow-up, seven (30%) hips from the AVN group had worsened clinical outcomes. A significant percentage of these hips were Kalamchi group IV. On the other hand, 16 hips had improved clinical outcomes and a significant percentage of them were Kalamchi group I. The difference in clinical outcomes between different AVN Kalamchi groups was found significant (P = 0.041). Contrary, at final follow-up, there was no significant difference between AVN Kalamchi groups in terms of radiological outcome categories (P = 0.12). Nevertheless, none of the hips with AVN Kalamchi groups III or IV ended with normal radiological outcomes, and none of AVN Kalamchi groups I, II, and III ended with unacceptable radiological outcomes.

Table 7 and Table 8 show the relationship between the final clinical and radiological outcomes with all variables for the study cohort. Significant variables from the univariate analysis for the relations of all variables with final clinical and radiological outcomes and the occurrence of AVN were entered into multivariate regression models. In the case of high multicollinearity, one or more variables were excluded from the model (Table 9). In the multivariate regression analysis, the presence of AVN, prolonged casting, and the need for a second surgery were strong determinants of unsatisfactory clinical outcomes (P = 0.002, 0.016, and 0.029 respectively). In another model, the presence of AVN, previous trials of closed reduction, and the need for a second surgery were significantly associated with worsening radiological outcomes. (P = 0.002, 0.032, and 0.013 respectively). In the last multivariate regression model, a previous trial of closed reduction, absence of OFH, prolonged casting, and the need for second surgery were strong risk factors for AVN (P = 0.001, < 0.001, 0.038, and 0.030 respectively).

**Table 7:** Relations of final clinical outcome with all variables for the study cohort IQR: Interquartile range, OR: Open Reduction, AP: Acetabuloplasty, FS: Femoral Shortening *:statistically significant p-value

Variable		Satisfactory n = 180	Unsatisfactory n = 9	P
Median (IQR)				
Age at surgery (months)		22 (18 – 28)	24 (22 – 31)	0.87
N (%)				
Previous trial of closed reduction	Yes	24 (13)	5 (56)	0.001*
	No	156 (87)	4 (44)	
Ossified Femoral Head	Yes	35 (19)	8 (89)	< 0.001*
	No	145 (81)	1 (11)	
Median (IQR)				
Operation time (min)		198 (155 – 298)	235 (182 – 315)	0.49
N (%)				
Surgical procedure	OR	34 (19)	3 (33)	0.87
	OR + AP	107 (59)	5 (56)	
	OR + AP + FS	39 (22)	1 (11)	
Blood transfusion	Yes	26 (14)	1 (11)	0.780
	No	154 (86)	8 (89)	
Median (IQR)				
Period of casting (days)		77 (69 – 90)	98 (75 – 163)	< 0.001*
N (%)				
Period of casting	Prolonged	53 (29)	6 (67)	0.019*
	Normal	127 (71)	3 (33)	
Complications	Yes	27 (15)	8 (89)	< 0.001*
	No	153 (85)	1 (11)	
Second surgery	Yes	16 (9)	7 (78)	< 0.001*
	No	164 (91)	2 (22)	
Physiotherapy	Yes	15 (8)	4 (44)	< 0.001*
	No	165 (92)	5 (56)	

**Table 8:** Relations of final radiological outcome with all variables for the study cohort IQR: Interquartile range, OR: Open Reduction, AP: Acetabuloplasty, FS: Femoral Shortening *: statistically significant p-value

Variable		Category 1 n = 153	Category 2 n = 34	Category 3 n = 2	P
Median (IQR)					
Age at surgery (months)		22 (17 – 27)	24 (20 – 35)	37 (29 – 46)	0.036*
N (%)					
Previous trial of closed reduction	Yes	17 (11)	11 (32)	1 (50)	0.003*
	No	136 (89)	23 (68)	1 (50)	
Ossified Femoral Head	Yes	16 (11)	25 (74)	0 (0)	< 0.001*
	No	137 (89)	9 (26)	2 (100)	
Median (IQR)					
Operation time (min)		194 (143 – 219)	226 (198 – 322)	286 (245 – 327)	0.039*
N (%)					
Surgical procedure	OR	29 (19)	8 (24)	0 (0)	0.23
	OR + AP	95 (62)	15 (44)	2 (100)	
	OR + AP + FS	29 (19)	11 (32)	0 (0)	
Blood transfusion	Yes	23 (15)	4 (12)	0 (0)	0.75
	No	130 (85)	30 (88)	2 (100)	
Median (IQR)					
Period of casting (days)		77 (52 – 90)	81 (69 – 98)	117 (95 – 138)	0.14
N (%)					
Period of casting	Prolonged	45 (29)	12 (35)	2 (100)	0.09
	Normal	108 (71)	22 (65)	0 (0)	
Complications	Yes	17 (11)	16 (47)	2 (100)	< 0.001*
	No	136 (89)	18 (53)	0 (0)	
Second surgery	Yes	8 (5)	13 (38)	2 (100)	< 0.001*
	No	145 (95)	21 (62)	0 (0)	
Physiotherapy	Yes	11 (7)	7 (21)	1 (50)	0.011*
	No	142 (93)	27 (79)	1 (50)	

**Table 9:** Regression Models AVN: Avascular Necrosis *: statistically significant p-value

-	Determinants	Odds ratio (95% CI)	P
Clinical outcome (Binary logistic)	AVN (Present)	1.17 (1.05 – 1.59)	0.002*
	Period of casting (days)	1.07 (1.02 – 1.19)	0.016*
	Second surgery (yes)	1.38 (1.07 – 5.66)	0.029*
Radiological outcome (Binary logistic)	AVN (Present)	1.14 (1.05 – 1.36)	0.002*
	Previous trial of closed reduction (exist)	1.27 (1.10 – 1.41)	0.032*
	Second surgery (yes)	1.49 (1.14 – 1.71)	0.013*
AVN (Binary logistic)	Previous trial of closed reduction (exist)	1.57 (1.12 – 4.30)	0.001*
	Ossified Femoral Head (non-ossified)	2.79 (2.03 – 5.99)	< 0.001*
	Period of casting (days)	1.13 (1.05 – 2.33)	0.038*
	Second surgery (yes)	1.48 (1.21 – 8.90)	0.030*

## Discussion

The incidence of AVN after DDH treatment varies depending on the method of treatment used. Previous studies have shown the incidence of AVN after open reduction ranging between 0 and 66% [3,16,20,21]. The risk of AVN after treatment of DDH has been the subject of several investigations. In response to the main objective of the current study, our results highlighted significant relations between the occurrence of AVN after open reduction of DDH and many different variables (Tables 2, 4). While our results emphasized the impact of strict DDH management protocol in our institute on the incidence and severity of AVN by overcoming probable AVN risk factors, still some risk factors were inevitable and couldn’t be averted such as previous trials of closed reduction, absent OFH, and presence of postoperative complications with subsequent revision surgeries.

There is general agreement that recommends early reduction of the dislocated hip in the first year of life [22-24]. Application of Pavlik harness or closed reduction followed by immobilization with spica cast is the standard of care in treating early diagnosed DDH, however, not all cases are successful and may require proceeding to an open reduction [25,26]. Arneill et al. (2021) in their initial 543 patients (644 hips) with DDH found 21 hips with AVN groups I-IV had open reduction after failure of closed reduction [1]. Their findings agree with our results which indicate a significant association of AVN with previous trials of closed reduction.

One of the protective factors for AVN occurrence following open or closed reduction for DDH is the presence of an OFH [20]. The results of our study agree with Bozkurt et al. (2022) who reported that the absence of an ossific nucleus at reduction time is one of the risk factors for developing AVN [10].

Open reduction remains at risk for many postoperative complications other than AVN. Razaq et al. (2016) investigated the postoperative complications of open reduction in patients with DDH to determine the possible risk factors associated with these complications. The most common complication observed was osteonecrosis (8.33%) followed by residual dysplasia and re-dislocation (5%), then infection (3.33%) [27]. Sankar et al. (2011) reported similar results and also noted in their findings that the most common causes for unsatisfactory outcomes were a large dysmorphic femoral head and abnormal femoral version [28]. After excluding stiffness which is in most instances a self-limited and expected complication after OR for DDH, the present study showed that AVN was the most common post-operative complication among our cohort (n=23 hips) followed by lateralization or re-dislocation (n=19) then persistent acetabular dysplasia (n=9). Our results showed a significantly increased incidence of AVN with the presence of other postoperative complications and of course the need for revision surgeries.

In the author’s institute, the management protocol when operating on DDH instructs to perform femoral shortening routinely in children older than two years of age and to avoid an unnecessarily lengthened time of hip spica. The protocol emphasizes providing sufficient inferior capsular release and performing adequate capsulorraphy and adequate pelvic osteotomy. Furthermore, the protocol recommends making all efforts to avoid failure of the initial open reduction and the need for revision surgery. All these issues were reported in many previous studies as risk factors that contribute to the increasing incidence of AVN after open reduction of DDH [29,30].

Jamil et al. (2022) reviewed 66 patients (76 hips) and divided them into two groups, group A (18 hips) had open reduction only, and group B (58 hips) had undergone open reduction and pelvic osteotomy. According to their findings, the majority of patients experienced satisfactory clinical (75.8%) and radiological (72.7%) outcomes [31]. Castañeda et al. (2018) reported that 80% of the operated hips in their study had good radiological outcomes, Severin grades I or II [21]. However, the results of the current study showed even higher satisfactory final clinical outcomes (95%) with normal and acceptable final radiological outcomes (Severin grades I, II and III) reaching 99% of all hips in our study cohort.

All children receiving treatment for hip dysplasia should be followed up and monitored radiologically for many years because of possible clinical deterioration or late-occurring complications [32]. Li et al. (2020) reported that 37.8% of patients with late-detected DDH who had undergone open reduction had poor hip function at the last follow-up appointment [33]. Our results agree with Dhar et al. (1990) who reported that age at surgery has no significant association with worsened clinical and radiological outcomes [34] and disagree with other studies that reported that worse outcomes were linked to late presentation and operating on older age group [33,35]. On the other hand, the results of our study support the findings of Gardner et al. (2014) which found that AVN was the main risk factor for a worsened outcome. They reported that the presence of AVN was seven times more likely to result in unsatisfactory radiological outcomes [36]. Likewise, our results showed a significant correlation between worsening clinical and radiological outcomes and with occurrence of AVN. Nevertheless, while the AVN group had a significant association with the final clinical outcome it did not correspond to the final radiological outcome.

This study has several limitations that warrant discussion. The retrospective nature of the study introduces potential bias, as data collection relied on existing medical records and documentation, which may not capture all relevant variables uniformly. Additionally, the single-center setting limits the generalizability of the findings; future studies adopting a multi-center approach could provide more robust and widely applicable results. The minimum follow-up duration of two years, while sufficient for initial evaluation, may not be adequate to assess late-onset complications or long-term outcomes comprehensively. Therefore, longer-term follow-up is strongly recommended for subsequent studies. A further limitation encountered during statistical analysis was the relatively small number of hips diagnosed with AVN, which reduced statistical power. Additionally, the presence of multicollinearity posed challenges, as several variables exhibited strong correlations with the occurrence of AVN and final outcomes. High multicollinearity was effectively managed by excluding one or more interrelated variables from the statistical model, but this approach may have omitted nuanced interactions among these factors. Despite these limitations, the study provides valuable insights into the management and outcomes of DDH.

Based on our findings, we encourage adopting early mobilization protocols and reducing casting duration to enhance recovery and minimize complications. We also encourage identifying high-risk patients early, particularly those without an ossified femoral head. Routine preoperative imaging to assess femoral head ossification could guide surgical planning and postoperative care. Tailored surgical strategies, including femoral shortening or adjustments to osteotomy techniques, may mitigate AVN risk in patients with significant preoperative deformities.

That being said, future studies and analyses should focus on generating stronger evidence by addressing our limitations that were mentioned earlier, increasing the number of studied cases, increasing the duration of the study, or adopting a multicenter approach.

## Conclusions

In conclusion, a previous trial of closed reduction, absence of an OFH, prolonged casting, and the need for a second surgery were strong risk factors for the occurrence of AVN. The current study highlighted the impact of AVN on final clinical and radiological outcomes after open reduction of DDH. This study also defined other factors which are strong determinants of unsatisfactory clinical and radiological outcomes. We recommend strict adherence to all measures that overcome these factors during all stages of surgical treatment of DDH to improve clinical outcomes in these patients.
